# *Blastocystis* occurrence and subtype diversity in European wild boar (*Sus scrofa*) from the Iberian Peninsula

**DOI:** 10.1186/s13567-024-01385-9

**Published:** 2024-10-07

**Authors:** Pamela C. Köster, Ana M. Figueiredo, Jenny G. Maloney, Alejandro Dashti, Begoña Bailo, Rita T. Torres, Carlos Fonseca, Atle Mysterud, Miguel Á. Habela, Antonio Rivero-Juarez, Joaquín Vicente, Emmanuel Serrano, Maria C. Arnal, Daniel Fernández de Luco, José A. Armenteros, Ana Balseiro, Guillermo A. Cardona, João Carvalho, Dário Hipólito, Joana Fernandes, Josman D. Palmeira, Rafael Calero-Bernal, David González-Barrio, Monica Santin, David Carmena

**Affiliations:** 1 Parasitology Reference and Research Laboratory, Spanish National Centre for Microbiology, Health Institute Carlos III, Majadahonda, Madrid Spain; 2 Faculty of Health Sciences, Alfonso X El Sabio University (UAX), Villanueva de la Cañada, Madrid, Spain; 3 Faculty of Medicine, Alfonso X El Sabio University (UAX), Villanueva de la Cañada, Madrid, Spain; 4 Department of Biology and CESAM, University of Aveiro, Aveiro, Portugal; 5 Centre for Ecological and Evolutionary Synthesis, Department of Bioscience, University of Oslo, Oslo, Norway; 6 Environmental Microbial and Food Safety Laboratory, Agricultural Research Service, United States Department of Agriculture, Beltsville, MD USA; 7 ForestWISE-Collaborative Laboratory for Integrated Forest & Fire Management, Vila Real, Portugal; 8 Department of Animal Health, Veterinary Sciences Faculty, Extremadura University, Caceres, Spain; 9 Infectious Diseases Unit, Maimonides Institute for Biomedical Research (IMIBIC), University Hospital Reina Sofía, University of Córdoba, Córdoba, Spain; 10 Center for Biomedical Research Network in Infectious Diseases (CIBERINFEC), Health Institute Carlos III, Madrid, Spain; 11 SaBio Group, Institute for Game and Wildlife Research, IREC (UCLM-CSIC-JCCM), Ciudad Real, Spain; 12 Wildlife Ecology & Health Group (WE&H), Wildlife Environmental Pathology Service (SEFaS), Department of Animal Medicine and Surgery, Autonomous University of Barcelona, Bellaterra, Spain; 13 Department of Animal Pathology, Veterinary Faculty, University of Zaragoza, Saragossa, Spain; 14 Council of Development, Territory Planning and the Environment of the Principado de Asturias, Oviedo, Spain; 15 Animal Health Department, Veterinary School, University of León, León, Spain; 16 Animal Health Department, Mountain Livestock Institute (CSIC-University of León), León, Spain; 17 Livestock Laboratory, Regional Government of Álava, Vitoria-Gasteiz, Spain; 18 Veterinary Biology Unit, Faculty of Veterinary Medicine, University of Zagreb, Heinzelova 55, 10000 Zagreb, Croatia; 19 Center for Evolutionary Hologenomics, The GLOBE Institute, University of Copenhagen, Copenhagen, Denmark; 20 SALUVET, Department of Animal Health, Faculty of Veterinary, Complutense University of Madrid, Madrid, Spain

**Keywords:** Epidemiology, NGS, subtype diversity wildlife, zoonoses, Spain, Portugal

## Abstract

The ongoing increase in wild boar populations across Europe has fostered human–wildlife conflicts, including the transmission of emerging pathogens with zoonotic importance. *Blastocystis* is a ubiquitous, faecal-oral transmitted protist that can cause gastrointestinal illnesses and is observed in humans and animals worldwide. The role of wildlife in the epidemiology of *Blastocystis* is insufficiently understood. Thus, we investigated the occurrence and subtype diversity of *Blastocystis* in free-ranging wild boars from the Iberian Peninsula using conventional PCR and next-generation amplicon sequencing of a fragment of the *ssu* RNA gene. A total of 459 wild boar faecal samples were collected across Spain (*n* = 360) and Portugal (*n* = 99) between 2014 and 2021. *Blastocystis* was present in 15.3% (70/459; 95% CI 12.1–18.9) of the wild boars analysed, and its occurrence was significantly higher in Portugal (34.3%, 34/99; 95% CI 25.1–44.6) than in Spain (10.0%, 36/360; 95% CI 7.1–13.6). Seven *Blastocystis* subtypes (ST5, ST10b, ST13–ST15, ST24b, and ST43) were detected among the surveyed wild boar populations, with greater variability detected in Portuguese samples. ST5 was identified in all the *Blastocystis*-positive animals, whereas 14.3% of them harboured ST mixed colonisations. Our results demonstrate that *Blastocystis* ST5 is particularly adapted to infect wild boars. The additional identification of zoonotic STs reinforces the role of wild boars as spreaders of zoonotic infections with public health significance.

**Supplementary Information:**

The online version contains supplementary material available at 10.1186/s13567-024-01385-9.

## Introduction

The wild boar (*Sus scrofa*) is widely distributed in Eurasia, from Europe to the Far East, including Southeast Asia, and extends as far as North Africa, South America, and the USA [1]. In Europe, a remarkable increase in wild boar populations has been recorded in the past four decades as a consequence of its high reproductive rate, supplementary feeding, lack of large predators, land abandonment, shrub encroachment, reduction in the number of human residents in rural areas, intensification of crop production, and changes from harsh to milder winters [2, 3]. Wild boars show a remarkable dispersion ability (more than 45 km on average) [4], colonising an astonishing variety of habitats ranging from the timberline to large cities [5]. Indeed, the wild boar is considered the second most abundant wild ungulate species in Europe, with more than three million individuals estimated [6, 7]. Overabundant and expanding wild boar populations increase human‒wildlife conflicts, including traffic accidents [8, 9], crop damage [10], threats to sensitive areas and species [11, 12], and the transmission of pathogens at the sylvatic‒domestic (including livestock and human) interface [13–15]. The current worldwide distribution and apparent population burgeoning and geographic expansion of wild boars have prompted the consideration of this species as a relevant potential source for emerging animal diseases (some of which are zoonotic), including animal tuberculosis (TB) [16, 17] and re-emerging African swine fever [18], Aujeszky’s disease virus [19], hepatitis E virus, bacteria (e.g., *Brucella* spp., *Erysipelothrix rhusiopathiae*), and parasitic infections [13, 14, 20].

The wild boar is one of the most hunted species in Europe, representing a potential source of zoonotic human infections, such as intestinal parasites, that are faecal- orally transmitted indirectly via ingestion of water or food contaminated with faecal material or directly via contact (through carcass handling) with infected animals. The increasing urbanisation of wild boar populations may also raise public health concerns about parasite cross-species transmission at the wild boar–domestic animal–human health interface. Among them, *Blastocystis*, a member of the Stramenopiles, is a ubiquitous protist that infects/colonises a broad range of human and nonhuman animal hosts [21]. Although *Blastocystis* is one of the most common microeukaryotes found in the human gastrointestinal tract [22], the clinical significance of *Blastocystis* is not fully understood. This protist has often been described as an asymptomatic coloniser in large human populations [23]. Furthermore, evidence from recent metagenomic studies suggests that *Blastocystis* may be part of the healthy gut microbiota in most circumstances [24–27].

*Blastocystis* is a highly pleomorphic microorganism with wide genetic diversity. On the basis of variability within the small subunit ribosomal RNA (*ssu* rRNA) gene, *Blastocystis* can be divided into 44 subtypes (STs) (ST1–ST17, ST21, and ST23–ST48) [28–33]. Among the 16 subtypes reported in humans, ST1 to ST4 are the most common, whereas ST5-ST10, ST12, ST14, ST16, ST23, ST35, and ST41 range from relatively uncommon to rare [34–39]. All other *Blastocystis* STs have been documented in non-human animal species thus far and are considered to have limited or negligible zoonotic potential. Because of the apparent loose host specificity of multiple *Blastocystis* STs, surveys investigating the prevalence and molecular diversity of protists from a variety of animal species and geographic origins are of interest to help disentangle the epidemiology and zoonotic potential of *Blastocystis* STs. This need is particularly evident for wild and domestic ungulate species, for which recent studies have demonstrated complex concomitant colonisation patterns involving up to 18 *Blastocystis* STs [33, 40–42] and variable associations between age groups and colonisation status [31]. These studies also highlighted the occurrence of cross-species transmission events of uncertain directionality that deserve further investigation.

Of particular interest is assessing intra- and inter-ST discrimination in host infection/colonisation and disease and ascertaining which animal hosts pose a risk to human infection and to what extent. Data on the epidemiology of *Blastocystis* in wild boar populations are relatively limited (Table 1) [43–57]. In this study, we analysed a large panel of faecal samples from free-ranging wild boars sampled in a broad Iberian geographic range covering Spain and Portugal.

**Table 1:** **Prevalence and molecular diversity of** ***Blastocystis*** **reported in wild boars (*Sus scrofa*) globally**.

Country	Population status	Prevalence (no. pos/total)	Detection method	Subtype(s) (*n*)	Mixed ST’s detected?	References
Brazil	Captive	13 (10/79)	CM	–	–	[43]
Brazil	Captive	77 (30/39)	PCR, SS	**ST1** (3), **ST4** (1), **ST5** (14), **ST8** (1)	No	[44]
China	Wild	0 (0/4)	PCR, SS	–	–	[45]
Iran	Wild	25 (3/12)	CM	–	–	[46]
Iran	Wild	44 (11/25)	CM	–	–	[47]
Italy	Wild	62 (26/42)	PCR, SS, NGS	**ST3**(1), **ST5** (10), ST15 (21)	Yes	[48]
Poland	Wild	50 (1/2)	PCR, SS	**ST5** (1)	No	[49]
Poland	Captive	80 (8/10)	PCR, SS	**ST5** (8)	No	[50]
Portugal	Wild	29 (42/144)	PCR, SS	**ST5** (42)	No	[51]
Portugal	Wild	34 (34/99)	PCR, NGS	**ST5** (34), **ST10a** (1), ST13 (1), **ST14** (1), ST15 (1), ST24b (1), ST43 (2)	Yes	This study
Slovakia	Captive	50 (1/2)	PCR, SS	**ST12** (1)	No	[52]
Slovakia	Wild	ND	PCR, SS	**ST15** (4), **ST10** (1)	ND	[53]
South Korea	Wild	10 (45/433)	PCR, SS	**ST5** (45)	No	[54]
Spain	Wild	0.7 (1/142)	PCR, SS	**ST5** (1)	No	[55]
Spain	Wild	10 (36/360)	PCR, NGS	**ST5** (22), ST15 (1)	Yes	This study
UK	Captive	50 (1/2)	PCR, SS	**ST5** (1)	No	[56]
UK	Captive	50 (2/4)	PCR, SS	**ST5** (2)	No	[57]

Subtypes previously reported in humans (regardless of their true zoonotic potential) are in bold.

CM: Conventional microscopy, ND: Not determined, NGS: Next-generation sequencing, PCR: Polymerase chain reaction, SS: Sanger sequencing.

## Materials and methods

### Sampling sites and sample collection

**Figure 1:**
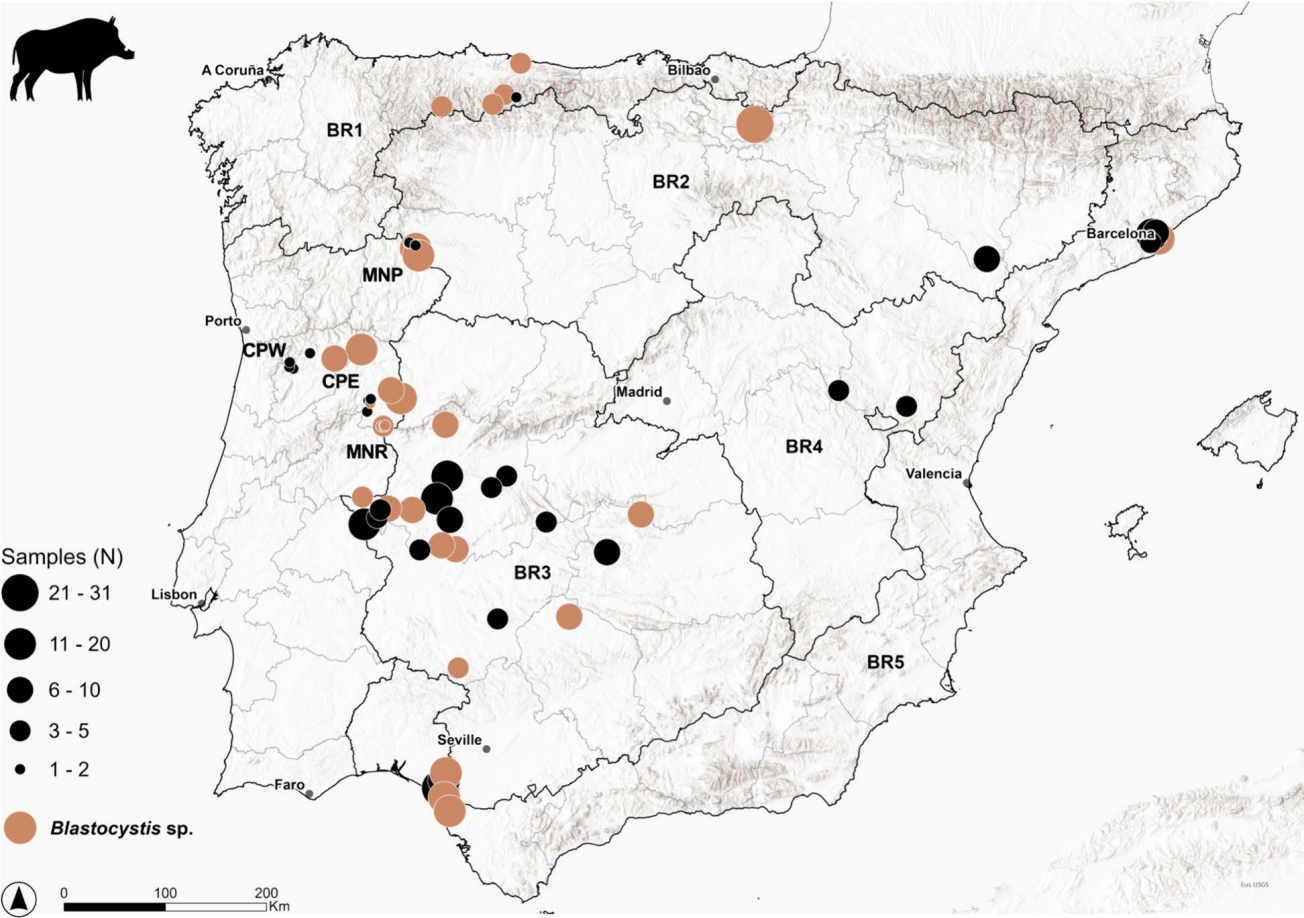
**Map of the Iberian Peninsula showing the sampled areas in Spain and Portugal and the geographical distribution of *Blastocystis* detected in wild boar (*Sus scrofa*).** BR2 encompasses Montesinho Natural Park (MNP), BR1 Central Portugal West (CPW), and BR3 Central Portugal East (CPS) and Malcata Nature Reserve (MNR).

Between autumn 2014 and summer 2021, a retrospective survey was performed in the Iberian Peninsula (Spain and Portugal). Faecal samples from wild boars collected throughout the five bioregions (BRs) of mainland Spain and three comparable BRs in Portugal were used for this purpose (Figure 1). A thorough description of the Spanish BRs can be found elsewhere [58, 59]. The main features of the three adapted Portuguese BRs sampled in the present study and the corresponding locations (MNP—Montesinho Natural Park, CPW—Central Portugal West, CPE—Central Portugal East and MNR—Malcata Nature Reserve) are summarised in Additional file 1 [60].

The sampling sites included hunting estates or game reserves, natural parks and other classified areas belonging to the European Union’s Natura 2000 Network sites [61]. Faecal samples were collected directly from the rectum of each animal during field necropsies after hunting or from the ground by prospecting several well-distributed transects representative of the different habitats throughout the sampling areas. For the latter case, samples were identified based on the morphology (e.g., content, shape, size) and deposition site by experienced and field-trained personnel. Faecal samples were placed in individually labelled sterile tubes, and collection dates and sites were recorded. Aliquots of these faecal samples were stored at −20 °C by each participating institution responsible for the sampling before being shipped to the Spanish National Centre for Microbiology (SNCM), Majadahonda (Spain), and the Department of Biology & CESAM, University of Aveiro (Portugal), for subsequent molecular analyses.

### DNA extraction and purification

Genomic DNA was isolated from approximately 200 mg of each wild boar faecal sample using the QIAamp DNA Stool Mini Kit (Qiagen, Hilden, Germany) according to the manufacturer’s instructions, except that samples mixed with InhibitEX buffer were incubated for 10 min at 95 °C. The extracted and purified DNA samples were eluted in 200 µL of PCR-grade water and stored at 4 °C until further molecular analysis. The DNA samples from the extractions carried out at the Department of Biology & CESAM facilities were then shipped to the SNCM for subsequent molecular testing.

### Molecular detection and characterisation of Blastocystis using Sanger sequencing

*Blastocystis* was initially identified via a direct PCR protocol targeting a fragment of the *ssu* rRNA gene of the parasite [62]. The assay uses the pan-*Blastocystis* barcode primer pair BhRDr (5ʹ-GAGCTTTTTAACTGCAACAACG-3ʹ) and RD5 (5ʹ-ATCTGGTTGATCCTGCCAGT-3ʹ) to amplify a PCR product of ~ 600 bp. The amplification reactions (25 µL) included 5 µL of template DNA and 0.5 µM of each primer. The amplification conditions consisted of one step at 95 °C for 3 min, followed by 30 cycles of 1 min each at 94 °C, 59 °C and 72 °C, with an additional 2 min final extension at 72 °C.

Amplicons of the expected size were sequenced in both directions by capillary DNA sequencing electrophoresis using BigDye® Terminator chemistry on an ABI PRISM 3130 automated DNA sequencer (Applied Biosystems, Foster City, CA, USA). The obtained consensus sequences were analysed using the Basic Local Alignment Search Tool (BLAST) for *Blastocystis* confirmation and subtype calling.

### Subtype identification using next-generation amplicon sequencing

Subsets of *Blastocystis* DNA samples whose *ssu*-PCR amplicons yielded bands of the expected size on agarose gels (regardless of Sanger sequencing confirmation) were shipped to the Environmental Microbial and Food Safety Laboratory, United States Department of Agriculture (Beltsville, Maryland, USA) for subsequent analyses. A next-generation amplicon sequencing (NGS) strategy was used to identify *Blastocystis* subtypes as previously described [40]. Briefly, a PCR using primers ILMN_Blast505_532F (5ʹ-TCGTCGGCAGCGTCAGATGTGTATAAGAGACAGGGAGGTAGTGACAATAAATC-3ʹ) and ILMN_Blast998_1017R (5ʹ-GTCTCGTGGGCTCGGAGATGTGTATAAGAGACAGTGCTTTCGCACTTGTTCATC-3ʹ (adapter sequences underlined) was used to amplify a fragment of the *ssu* rRNA gene (ca. 500 bp). These primers were identical to Blast505_532F/Blast998_1017R [63], except that they contained Illumina overhang adapter sequences at the 5′ end. The final libraries were quantified via Qubit fluorometric quantitation (Invitrogen, Carlsbad, CA, USA) before normalisation. A final pooled library concentration of 8 pM with a 20% PhiX control was sequenced using an Illumina MiSeq and a 600 cycle v3 kit (Illumina, San Diego, CA, USA). Paired-end reads were processed and analysed with an in-house pipeline as previously described [40]. The raw FASTQ files were submitted to the NCBI sequence read archive under project number PRJNA1022431. The nucleotide sequences obtained in this study have been deposited in GenBank under the accession numbers OR730909–OR730919, OR730924, OR730933, OR730938, and OR730943–OR730947.

### Data analysis

Parasite prevalence was estimated using a binomial test in R software [64], establishing confidence limits with 95% intervals (CI) and χ^2^ values with the chi-square test function.

## Results

### Occurrence of *Blastocystis*

A total of 459 faecal samples were collected across Spain (*n* = 360) and Portugal (*n* = 99) between 2014 and 2021 (Additional file 2). Overall, 15.3% (70/459; 95% CI 12.1–18.9) of the faecal samples from the wild boar analysed were confirmed to be positive for *Blastocystis* by Sanger sequencing and/or next-generation sequencing (NGS). Samples that yielded PCR amplicons of the expected size but could not be confirmed by Sanger sequencing and/or NGS were conservatively considered negative. Wild boars from Portugal presented higher *Blastocystis* carriage rates (34.3%, 34/99; 95% CI 25.1–44.6) than those from Spain did (10.0%, 36/360; 95% CI 7.1–13.6), and this difference was statistically significant [χ^2^ (1, *n* = 459) = 22.1, *P* < 0.001].

Table 2 shows the distribution of *Blastocystis* in wild boars from Spain according to the sampling variables considered. The occurrence of protists varied greatly among bioregions [χ^2^ (4, *n* = 360) = 23.0, *P* < 0.001], with animals from BR1 (38.1%) and BR2 (23.1%) having the highest prevalence. All eight animals available from BR4 tested negative for *Blastocystis*. At the sampling site, wild boars from game reserves were more likely to harbour *Blastocystis* [χ^2^ (3, *n* = 360) = 16.8, *P* < 0.001]. *Blastocystis* presence was significantly greater (34.6%) in wild boars sampled in 2014 [χ^2^ (4, *n* = 360) = 18.9, *P* < 0.001], all from the province of Álava in BR2 (Additional file 2).

**Table 2:** **Prevalence of** ***Blastocystis*** **subtypes in Spanish wild boars (*n*** **= 360) according to the bioregion of origin, type of sampling site, and sampling year**.

Variable	Samples (*n*)	*Blastocystis*-positive (*n*)^a^	*Blastocystis*-positive (%)	95% CI (%)	*P* value	Subtypes detected^b^ (*n*)
Bioregion					**< 0.001**	
BR1	21	8	38.1	18.1–61.6		**ST5 **(8)
BR2	39	9	23.1	11.1–39.3		**ST5** (7), **ST5**/ST15 (1)
BR3	150	13	8.7	4.7–14.4		**ST5** (2)
BR4	8	0	0.0	–		–
BR5	142	6	4.2	1.6–9.0		**ST5 **(4)
Type of sampling site					**< 0.001**	
Hunting state	197	22	11.2	7.1–16.4		**ST5** (9), **ST5**/ST15 (1)
Game reserve	21	8	38.1	18.1–61.6		**ST5** (8)
Natural protected area	97	4	4.1	1.1–10.2		**ST5** (4)
Urban/suburban	45	2	4.4	0.5–15.2		Not available
Sampling year^c^					**< 0.001**	
2014	26	9	34.6	17.2–55.7		**ST5** (7), **ST5**/ST15 (1)
2018	148	6	4.1	1.5–8.6		**ST5** (4)
2019	52	6	11.5	4.3–23.4		**ST5** (2)
2020	70	5	7.1	2.4–15.9		Not available
2021	60	9	15.0	7.1–26.6		**ST5 **(7)

95% confidence intervals (95% CI) are included. The values in bold represent statistical significance and subtypes previously reported in humans (regardeless of their true zoonotic potential).

^a^Samples were considered positive when *Blastocystis* was identified after Sanger and next-generation sequencing.

^b^Subtype information is only included for the 22 samples in which next-generation amplicon sequencing was conducted.

^c^Four samples from unknown sampling years, with one of the samples positive for *Blastocystis* ST5.

Table 3 shows the distribution of *Blastocystis* in wild boars from Portugal according to the sampling variables considered. All the investigated animals were from naturally classified areas. Neither the bioregion [χ^2^ (2, *n* = 99) = 1.3, *P* = 0.515] nor the sampling year [χ^2^ (2, *n* = 99) = 2.0, *P* = 0.373] influenced the occurrence of the protist in the investigated wild boar subpopulation.

**Table 3:** **Prevalence of** ***Blastocystis* in Portuguese wild boars (*n*** **= 99) according to the bioregion of origin and sampling year**.

Variable	Samples (*n*)	*Blastocystis* positive (*n*)^a^	*Blastocystis* positive (%)	95% CI (%)	*P* value	Subtypes detected^b^ (*n*)
Bioregion					0.515	
BR1	12	3	25.0	5.5–57.2		**ST5** (3)
BR2	39	9	23.1	11.1–39.3		**ST5** (6), **ST5**/**ST10a** (1), **ST5**/**ST14** (1), **ST5**/ST43 (1)
BR3	48	22	45.8	31.4–60.8		**ST5** (18), **ST5**/**ST13** (1), **ST5**/ST15 (1), **ST5**/ST24b (1), **ST5**/ST43 (1)
Sampling year					0.373	
2019	64	18	28.1	17.6–40.8		**ST5** (14), **ST5**/**ST10a** (1), **ST5**/**ST14** (1), **ST5**/ST24b (1), **ST5**/ST43 (2)
2020	21	8	38.1	18.1–61.6		**ST5** (8)
2021	14	8	57.1	28.9–82.3		**ST5** (6), **ST5**/**ST13** (1), **ST5**/ST15 (1)

All the samples were collected from naturally classified areas. 95% confidence intervals (95% CI) are included. BR1 encompasses Central Portugal West (CPW), BR2 Montesinho Natural Park (MNP), and BR3 Central Portugal East (CPE) and Malcata Nature Reserve (MNR). Subtypes previously reported in humans (regardless of their true zoonotic potential) are in bold.

^a^Samples were considered positive when *Blastocystis* was identified after Sanger and next-generation sequencing.

^b^Subtype information obtained from all positive samples via next-generation amplicon sequencing.

### Molecular characterisation of *Blastocystis*

Among the 36 confirmed *Blastocystis* samples from wild boars in Spain, 31 were identified as ST5 by Sanger sequencing. Seventeen of them, plus five dubious samples (i.e., samples for which no Sanger sequencing data were obtained due to insufficient or poor-quality DNA), were subsequently analysed by NGS. Two *Blastocystis* subtypes (ST5 and ST15) were found by NGS among the 22 *Blastocystis*-positive samples analysed (Table 2 and Additional file 2). ST5 was the most prevalent *Blastocystis* ST identified (100%, 22/22) in this wild boar subpopulation, whereas ST15 was found in a single (4.5%, 1/22) isolate as a mixed coloniser with ST5 (Additional file 2).

Among the 34 confirmed *Blastocystis* samples from wild boars from Portugal, 24 were identified by Sanger sequencing as ST5. All of them, plus ten dubious samples for which no Sanger sequencing data were obtained, were subsequently analysed by NGS. Seven STs (ST5, ST10a, ST13, ST14, ST15, ST24b, and ST43) were identified among the 34 *Blastocystis*-positive samples (Table 3 and Additional file 2). Similar to the wild boar population from Spain, ST5 was the most prevalent ST identified in this subpopulation (100%, 34/34), followed by ST43 (5.9%, 2/34). The remaining STs identified (ST10a, ST13, ST15, ST15, and ST24b) were only rarely found (2.9% each, 1/34) and were always a mixed colonisation with ST5 (Table 3 and Additional file 2).

### Blastocystis intra‑subtype diversity by NGS

Intra-subtype diversity was observed in only two STs, ST5 and ST15, the latter found in both Spain and Portugal, infecting one specimen each. No intra-subtype variability was detected within ST10a, ST13, ST14, ST24b, or ST43, where a single genetic variant was identified (Table 4). ST5 had the highest intra-subtype diversity, with eight unique genetic variants among the 56 *Blastocystis*-positive samples belonging to this ST. Four of them represented genetic variants shared between the Spanish and Portuguese populations. The remaining four were exclusively found circulating within the Spanish or Portuguese subpopulations (two each; Table 4 and Additional file 2).

**Table 4:** **Diversity of *Blastocystis* subtypes and unique genetic variants observed using next-generation amplicon sequencing (NGS) among *Blastocystis*-positive wild boars from Spain (*n*** **= 22) and Portugal (*n*** **= 34)**.

Subtype	Subgroup	Samples (*n*)	Unique genetic variants (*n*)	Frequency of positive simples (%)	GenBank accession number(s)^a^
**ST5**		56	8	100	OR730909(P)/OR730910(S)
					OR730911(P)/OR730912(S)
					OR730916(P)/OR730917(S)
					OR730918(P)/OR730919(S)
					OR730933(S)
					OR730938(S)
					OR730943(S)
					OR730947(P)
**ST10**	**ST10a**	1	1	1.8	OR730914(P)
ST13		1	1	1.8	OR730913(P)
**ST14**		1	1	1.8	OR730946(P)
ST15		2	2	3.6	OR730944(S)
					OR730945(P)
ST24	ST24b	1	1	1.8	OR730915(P)
ST43		2	1	3.6	OR730924(P)

Subtypes previously reported in humans (regardless of their true zoonotic potential) are in bold.

^a^ For unique variants identified in Spain and Portugal, two sequences were submitted to GenBank. The country in which the sequences were identified is denoted in parentheses by the GenBank accession number. P and S denote Portugal and Spain, respectively.

### Mixed ST colonisations discriminated by NGS

Among the 56 positive samples sequenced in the present study via NGS, only 8 (14.3%) contained a co-colonisation encompassing ST5 with another *Blastocystis* ST. Mixed colonisations in wild boars from Spain were found in only one sample (4.5%, 1/22; Table 2 and Additional file 2), whereas they appeared to be more common in wild boars from Portugal (20.6%, 7/34; Table 3 and Additional file 2). *Blastocystis* colonisation by a single ST always involved ST5 regardless of the origin of the sampled animal (Additional file 2). The only wild boar from Spain harbouring a *Blastocystis* mixed ST colonisation presented ST5 and ST15. The seven Portuguese wild boars harbouring *Blastocystis* mixed ST colonisations presented up to seven distinct STs (ST5, ST10a, ST13, ST14, ST15, ST24b, and ST43) in six combinations (Table 3 and Additional file 2). However, in both countries, mixed colonisations encompassing two subtypes primarily carry ST5 (99.6–99.8%), whereas the remaining six STs were detected at residual (0.1–0.4%) carriage rates (Table 5).

**Table 5:** **Prevalence of *Blastocystis*** **subtype/subgroups and the means and ranges of subtype/subgroups detected in wild boars from Spain (SP) (*n*** **= 22) and Portugal (PT) (*n*** **= 34) using next-generation amplicon sequencing (NGS) in the present study**.

Subtype	Subtype prevalence (%)		Subtype reads (mean, %)		Subtype reads (range, %)	
	SP wild boar	PT wild boar	SP wild boar	PT wild boar	SP wild boar	PT wild boar
**ST5**	100	100	100	100	99.6–100	99.8–100
**ST10a**	0	2.9	–	0.2	–	0.2
ST13	0	2.9	–	0.1	–	0.1
**ST14**	0	2.9	–	0.1	–	0.1
ST15	4.5	2.9	0.4	0.1	0.4	0.1
ST24b	0	2.9	–	0.1	–	0.1
ST43	0	5.9	–	0.2	–	0.1–0.2

Subtypes previously reported in humans (regardless of their true zoonotic potential) are in bold.

## Discussion

This survey represents the largest attempt to assess the occurrence, molecular diversity, and zoonotic potential of *Blastocystis* subtypes in wild boars conducted in the Iberian Peninsula to date. Our study has several strengths, including a large sample size, broad geographic coverage, the use of highly sensitive molecular methods for detecting and discriminating *Blastocystis* genetic variants, and the assessment of the presence of mixed STs within a sample. The survey is also timely because information on the contribution of wild boar to *Blastocystis* epidemiology is scarce [21, 65] (Table 1). This ubiquitous protist has been detected in a wide range of domestic and wild animals, suggesting the potential for zoonotic transmission events in both directions (animal → human and human → animal) [66–71]. In Europe, prevalence rates in wild boars have been reported, ranging from 1–62% in free-living animals and 50–80% in captive animals. Globally, most of the *Blastocystis* cases documented in wild boars reported ST5 (79.7%, 184/231) (Table 1). ST5 is also the most widely reported ST in surveys conducted domestically [21], suggesting that this subtype is particularly well adapted to colonise members of the Suidae family. Our data revealed an overall *Blastocystis* colonisation rate of 15.3% in wild boars, with higher rates in wild boars from Portugal (34.3%) than in their counterparts from Spain (10.0%). These figures align with those estimated in a recent national study conducted in Portugal (29.0%, 42/144) [51]. However, a lower presence of *Blastocystis* (0.7%, 1/142) was detected in wild boar faeces in southern Spain [55].

In our study, NGS analyses confirmed the occurrence of seven distinct *Blastocystis* STs, including subgroup variants of ST10 and ST24 (ST5, ST10a, ST13, ST14, ST15, ST24b, and ST43), which circulate within the surveyed wild boar populations, with greater variability (in terms of genetic diversity and mixed STs colonisation rates) in wild boars from Portugal than in those from Spain. In addition to ST5, the remaining subtypes were missed by Sanger sequencing. While in Spain, only 4.5% (1/22) of the *Blastocystis*-positive wild boars identified by NGS harboured mixed colonisations, a much higher co-colonisation rate (20.6%, 7/34) was observed in their Portuguese counterparts. The reason for the higher prevalence and genetic variability rates observed in Portugal is unclear. Cross-species transmission involving other wildlife species (e.g., cervids) does not seem to be a plausible explanation, as no differences in the distribution of free-living species and management practices of natural protected/classified areas exist between the surveyed Spanish and Portuguese regions. However, free-roaming livestock herds can potentially act as local sources of *Blastocystis* in areas where sylvatic and domestic transmission cycles overlap. Indeed, in a parallel study targeting the same areas sampled in the present study, *Blastocystis* prevalence rates ranging from 56–80% were reported among cattle, sheep, and goats, and 22 distinct *Blastocystis* STs (including the ST10, ST24, and ST42 subgroups) were identified: ST1–ST3, ST5–ST7, ST10/b, ST13, ST14, ST21, ST23, ST24a/b/c, ST25, ST26, ST30, ST42a/b, ST43, and ST44 [42]. Similarly, cattle from Spain have been demonstrated to harbour up to 10 *Blastocystis* subtypes, including ST1, ST3, ST5, ST10, ST14, ST21, ST23, ST24, ST25, and ST26 using also NGS [41]. Taken together, these data might indicate that the presence (at low or very low rates) of *Blastocystis* STs other than ST5 in Iberian wild boars is the direct consequence of sporadic spillover events from livestock (primarily cattle) sharing habitats, most likely through environmental faecal contamination of water or grass fields. In fact, in addition to ST15 (also reported in Spanish wild boars), all *Blastocystis* STs identified in Portuguese wild boars were previously reported in livestock species from Portugal [42]. Cross-species transmission at the domestic‒wildlife interface has been previously demonstrated for other pathogens, such as *Coxiella burnetii* [72]. Additionally, supplemental feeding is a common practice in hunting states and game reserves in Mediterranean habitats and is usually related to the maintenance of artificial high population densities. This practice is known for increasing disease transmission risk in wildlife due to aggregation behaviours. However, it can also be used as a wildlife disease management option by delivering vaccines or anti-parasitic agents throughout the feed [73], which could explain the low *Blastocystis* prevalence and genetic diversity found in Spanish wild boars.

Our results revealed that wild boars in the Iberian Peninsula are suitable reservoirs for seven distinct *Blastocystis* STs (ST5, ST10a, ST13, ST14, ST15, ST24b, and ST43), of which ST5, ST10, and ST14 are potentially zoonotic. ST5 is the most prevalent ST reported in wild boar and domestic pigs worldwide, suggesting that swine are its natural host. Thus, ST5 has been detected in all but two of the studies that conducted *Blastocystis* subtyping in wild boars (Table 1). ST5 in wild boar has been documented in Brazil, Italy, Poland, Portugal, South Korea, Spain, and the United Kingdom (Table 1). Subtypes other than ST5 have also been detected in this host, including ST15 in wild boar faecal samples from Italy and Slovakia, as well as potentially zoonotic STs, including ST1, ST3, ST4, ST8, and ST10 [65] (Table 1). The presence of genetically diverse subtypes, representing differences in parasite-host preference, zoonotic potential, pathogenesis, and probably clinical manifestations, is another important issue associated with *Blastocystis* carriage. Human cases are primarily due to infection/colonisation by ST1–ST4; however, at least 12 additional STs (ST5–ST10, ST12, ST14, ST16, ST23, ST35, and ST41) have also been reported in human samples with varying frequencies [34–39]. From the “One Health” perspective, which links human, animal, and environmental health, a threat to any of the components of this triad can substantially impact the others [74]. Consequently, the probable presence of potential zoonotic *Blastocystis* STs in wild boars can influence humans and other animal species that share the same habitat.

This study had potential limitations that may have biased, at least partially, the results obtained. First, its retroactive nature required that some of the analysed faecal samples be stored at −20 °C for up to seven years before DNA extraction and molecular testing. Long-term storage may have altered the quantity/quality of parasite DNA, compromising the performance of the PCRs used for diagnostic and genotyping purposes. Second, owing to the legal hunting periods, our opportunistic sampling strategy limited our ability to capture potential seasonal variations in *Blastocystis* occurrence in wild boars. Third, the conventional PCR used for screening purposes lacks inhibition control. It is possible that an unknown number of our allegedly *Blastocystis*-negative samples indeed inhibited the PCR. Fourth, even though the sampling carried out in Spain was conducted nationwide, in Portugal, it was carried out only in the northeast and central areas of the country, taking advantage of ongoing projects, meaning that the results may not reflect the whole Portuguese scenario. Clearly, more research with a proper design should be conducted to disentangle how environmental, host, and management factors can modulate the risk of exposure of wild boar to *Blastocystis*.

This is the largest molecular epidemiological study investigating the presence and genetic diversity of *Blastocystis* in wild boars conducted in the Iberian Peninsula to date. Overall, the presence of *Blastocystis* was relatively low (10%) in wild boars from Spain and was caused mainly by swine-adapted ST5. The opposite scenario was found in Portugal, with a much higher prevalence (34.3%) and genetic diversity (up to 7 STs), indicative of possible cross-species transmission or contamination from free-ranging livestock animals that share habitats. Our results show that wild boars, which are most likely in contact with domestic ungulates and possibly other wild animals, are important reservoirs of *Blastocystis* in the Iberian Peninsula. However, spurious infections (e.g., those expected in highly anthropized environments such as agricultural and peri-urban areas) cannot be ruled out. In this sense, adopting regular monitoring programs, encompassing the sampling of both wild and domestic animals, with more extensive national coverage and sampling sites, involving hunting associations and other partners (universities, national labs) to increase sample collection and storage, may help us obtain a better picture of the *Blastocystis* epidemiological scenario in the Iberian Peninsula, as well as a wide array of other protists and zoonoses, and its potential transmission risks for the human compartment.

## Supplementary Information

** Additional file 1. Summary of the sampling sites in Portugal according to bioregion with an emphasis on environmental, wildlife and flora features, adapted from PNVSFS (2020) and Muñoz et al. **[58]**.** The numbers of wild boar faecal samples collected at each location are indicated.

**Additional file 2. Full dataset showing the epidemiological data used in the analyses conducted in this study, as well as the diagnostic and molecular results obtained**.

## Data Availability

The data that support the findings of this study are available within the main body of the manuscript and its supplementary material.
