# Correction: *Blastocystis* occurrence and subtype diversity in European wild boar (*Sus scrofa*) from the Iberian Peninsula

**DOI:** 10.1186/s13567-024-01408-5

**Published:** 2024-11-14

**Authors:** Pamela C. Köster, Ana M. Figueiredo, Jenny G. Maloney, Alejandro Dashti, Begoña Bailo, Rita T. Torres, Carlos Fonseca, Atle Mysterud, Miguel Á. Habela, Antonio Rivero-Juarez, Joaquín Vicente, Emmanuel Serrano, Maria C. Arnal, Daniel Fernández de Luco, José A. Armenteros, Ana Balseiro, Guillermo A. Cardona, João Carvalho, Dário Hipólito, Joana Fernandes, Josman D. Palmeira, Rafael Calero-Bernal, David González-Barrio, Monica Santin, David Carmena

**Affiliations:** 1 Parasitology Reference and Research Laboratory, Spanish National Centre for Microbiology, Health Institute Carlos III, Majadahonda, Madrid Spain; 2 Faculty of Health Sciences, Alfonso X El Sabio University (UAX), Villanueva de la Cañada, Madrid, Spain; 3 Faculty of Medicine, Alfonso X El Sabio University (UAX), Villanueva de la Cañada, Madrid, Spain; 4 Department of Biology and CESAM, University of Aveiro, Aveiro, Portugal; 5 Centre for Ecological and Evolutionary Synthesis, Department of Bioscience, University of Oslo, Oslo, Norway; 6 Environmental Microbial and Food Safety Laboratory, Agricultural Research Service, United States Department of Agriculture, Beltsville, MD USA; 7 ForestWISE-Collaborative Laboratory for Integrated Forest & Fire Management, Vila Real, Portugal; 8 Department of Animal Health, Veterinary Sciences Faculty, Extremadura University, Caceres, Spain; 9 Infectious Diseases Unit, Maimonides Institute for Biomedical Research (IMIBIC), University Hospital Reina Sofía, University of Córdoba, Córdoba, Spain; 10 Center for Biomedical Research Network in Infectious Diseases (CIBERINFEC), Health Institute Carlos III, Madrid, Spain; 11 SaBio Group, Institute for Game and Wildlife Research, IREC (UCLM-CSIC-JCCM), Ciudad Real, Spain; 12 Wildlife Ecology & Health Group (WE&H), Wildlife Environmental Pathology Service (SEFaS), Department of Animal Medicine and Surgery, Autonomous University of Barcelona, Bellaterra, Spain; 13 Department of Animal Pathology, Veterinary Faculty, University of Zaragoza, Saragossa, Spain; 14 Council of Development, Territory Planning and the Environment of the Principado de Asturias, Oviedo, Spain; 15 Animal Health Department, Veterinary School, University of León, León, Spain; 16 Animal Health Department, Mountain Livestock Institute (CSIC-University of León), León, Spain; 17 Livestock Laboratory, Regional Government of Álava, Vitoria-Gasteiz, Spain; 18 Veterinary Biology Unit, Faculty of Veterinary Medicine, University of Zagreb, Heinzelova 55, 10000 Zagreb, Croatia; 19 Center for Evolutionary Hologenomics, The GLOBE Institute, University of Copenhagen, Copenhagen, Denmark; 20 SALUVET, Department of Animal Health, Faculty of Veterinary, Complutense University of Madrid, Madrid, Spain

**Correction:** **Veterinary Research (2024) 55:133 **10.1186/s13567-024-01385-9

Following publication of the original article [1], the authors updated the Acknowledgements section to include the code of the COST Action.

The incorrect Acknowledgements is:

This work is based upon work from COST Action Blastocystis under One Health, supported by COST (European Cooperation in Science and Technology). Sampling in the Basque Country was conducted by members of the Association for the Defence of the Game Natural Heritage of the Basque Country (ARTIO). We thank the Dirección General del Medio Natural y Planificación Rural del Principado de Asturias (Oviedo, Spain). We thank Aleksey Molokin and Nadja S. George for their technical assistance. Wildlife Ecology & Health Group (WE&H): The following investigators (in alphabetic order) participated in the Wildlife Ecology & Health Group (WE&H): Carles Conejero, Carlos González-Crespo, Cristina Garrido, Diana Gassó, Emmanuel Serrano, Gregorio Mentaberre, Irene Torres-Blas, Josep Estruch, Josep Pastor, Jorge Ramón López-Olvera, María Escobar-González, Marta Valldeperes, Montse Mesalles, Rafaela Cuenca, Roser Velarde, Santiago Lavín.

The correct Acknowledgements is:

This publication is based upon work from COST Action *Blastocystis* under One Health, CA21105, supported by COST (European Cooperation in Science and Technology). Sampling in the Basque Country was conducted by members of the Association for the Defence of the Game Natural Heritage of the Basque Country (ARTIO). We thank the Dirección General del Medio Natural y Planificación Rural del Principado de Asturias (Oviedo, Spain). We thank Aleksey Molokin and Nadja S. George for their technical assistance. Wildlife Ecology & Health Group (WE&H): The following investigators (in alphabetic order) participated in the Wildlife Ecology & Health Group (WE&H): Carles Conejero, Carlos González-Crespo, Cristina Garrido, Diana Gassó, Emmanuel Serrano, Gregorio Mentaberre, Irene Torres-Blas, Josep Estruch, Josep Pastor, Jorge Ramón López-Olvera, María Escobar-González, Marta Valldeperes, Montse Mesalles, Rafaela Cuenca, Roser Velarde, Santiago Lavín.

The original article has been corrected.

## References

[1] Köster PC, Figueiredo AM, Maloney JG, Dashti A, Bailo B, Torres RT, Fonseca C, Mysterud A, Habela MA, Rivero-Juarez R, Vicente J, Serrano E, Arnal MC, de Luco DF, Armenteros JA, Balseiro A, Cardona GA, Carvalho J, Hipólito D, Fernandes J, Palmeira JD, Calero-Bernal R, González-Barrio D, Santin M, Carmena D (2024) *Blastocystis* occurrence and subtype diversity in European wild boar (*Sus scrofa*) from the Iberian Peninsula. Vet Res 55:133. 10.1186/s13567-024-01385-9 39375799 10.1186/s13567-024-01385-9 PMC11460206

