# Supplementary material for: Blastocystis occurrence and subtype diversity in European wild boar (Sus scrofa) from the Iberian Peninsula
Source: Vet Res. 2024 Oct 7;55:133. doi: 10.1186/s13567-024-01385-9 (PMC11460206; doi:10.1186/s13567-024-01385-9)
Supplement: Supplementary file 1 — Additional file 1. Summary of the sampling sites in Portugal according to bioregion with an emphasis on environmental, wildlife and flora features, adapted from PNVSFS (2020) and Muñoz et al. [58]. The numbers of wild boar faecal samples collected at each location are indicated. [file 13567_2024_1385_MOESM1_ESM.docx]

**Additional file 1 Summary of the sampling sites in Portugal according to bioregion with an emphasis on environmental, wildlife and flora features, adapted from PNVSFS (2020) and Muñoz et al. [58].** Numbers of wild boar faecal samples collected in each location are indicated.

| **Location** | **Bioregion** | **Environmental features** | **Wildlife and domestic animal’s^1^ features** | **Sampling site features** |
| --- | --- | --- | --- | --- |
| Montesinho Natural Park (MNP) | BR2 | Continental Mediterranean climate. Dry, hot summers, dry, cold winters. Open, cereal landscapes with pine or oak woodlands, limited to the north by mountains | Wild boar abundant. Other ungulate species: red deer and roe deer widely distributed. Cattle *n* = 4816; goat *n* = 1717; sheep *n* = 34 773; pig *n =* 1153. | Samples collected: 41. Oak and chestnut forests (e.g., *Quercus pyrenaica*, *Castanea sativa*) and shrub vegetation (e.g., *Ulex europaeus*, *Cistus landanifer*). AR: 438–1,481; MAP: 1200; MAT: 9.0 |
| Central Portugal West (CPW) | BR1 | Atlantic climate with high precipitation rates. Pastures and deciduous woodlands. Mountainous habitats | Wild boar abundant. Other ungulate species: locally roe deer recently reintroduced. Cattle *n* = 555; goat *n =* 229; sheep *n =* 4064; pig *n =* 16. | Samples collected: 14. Woodlands (e.g., *Quercus robur*, *Pinus pinaster*) and shrubs (e.g., *Ulex* spp., *Erica* spp., *Pterospartum tridentatum*). AR: 800–1,381; MAP: 2023; MAT 9.8 |
| Central Portugal East (CPE) | BR3 | Continental thermo Mediterranean climate. Pastures and crops with interspersed vegetation, sometimes forming savannah-like structures. Low-altitude mountains with scrubland | Wild boar abundant. Other ungulate species: roe deer widely distributed, and red deer with limited distribution. Cattle *n =* 7700; goat *n* = 1643; sheep *n* = 19 810; pig *n* = 6115. | Samples collected: 39. Scrublands (e.g., *Cistus* spp., *Juniperu*s spp.) and tree species (e.g., *Quercus. rotundifolia*, *Quercus pyrenaica*). AR: 700–1,000. MAP: 1,195; MAT: 10.5 |
| Malcata Nature Reserve (MNR) | BR3 | Continental thermo Mediterranean climate. Pastures and crops with interspersed vegetation, sometimes forming savannah-like structures. Low altitude mountains with scrubland | Wild boar abundant. Other ungulate species: roe deer, widely distributed, and red deer with limited distribution. Cattle *n =* 7663; goat *n* = 2873; sheep *n* = 63 736; pig *n =* 3208. | Samples collected: 10. Scrublands (e.g., *Cistus* spp., *Erica* spp.) and tree species (e.g., *Quercus pyrenaica*, *Quercus suber*). AR: 425–1,078; MAP: 849; MAT 15.7 |

^1^Number livestock animals across the locations (MNP, CPW, CPE and MNR). Numbers corresponding to the 2017 data provided by the Direção Geral de Alimentação e Veterinária (DGAV) [60]. Insufficient data to determine animals raised under intensive and extensive systems.

AR, Altitude range, in metres; MAP, Mean annual precipitations, in millimetres; MAT, Mean annual temperature, in Celsius degrees.
